# Retirement Planning: Reimagined and Revamped for Successful Aging

**DOI:** 10.1093/geroni/igaa057.118

**Published:** 2020-12-16

**Authors:** Rajiv Nagaich, Carol Redfield, Ben Harvill, Scott Schill

**Affiliations:** 1 Aging Options, Federal Way, Washington, United States; 2 Aging Options, Seattle, Washington, United States

## Abstract

What grade should current retirement planning programs receive in which thousands of Americans participated in for their third segment of life? If success is measured by basic goal achievements that most desire (i.e. financial security, not be a burden, and avoiding institutional living), then we have failed significantly. Ninety percent of the population want to age in their own home, yet, only 30% get to. Sixty-six percent report that their employers did “nothing” to help with retirement transition; another 16% state “not sure” to what employers actually did. Seventy percent will travel through Long Term Care services in their lifetime, yet only 8% have LTC insurance. Only one-fourth of Baby Boomers are confident that they will have enough money to last through retirement. Retirement contribution starts with the first paycheck followed by 3-4 decades of employment with possible access to retirement preparedness counsel. Why, then, has planning fall short of being adequate? Are individuals too trusting of the programs from their employers or financial advisors that they don’t question completeness? Are professionals practicing in siloed spaces that they fail to realize their services may impact client’s other areas of life, especially, last years of life? Why is there a lack in a multi-disciplinary team approach which can ensure better predictability of outcomes? We propose a multi-pronged, integrated program: LifePlanning Model, which includes a coordinated, multi-disciplinary approach with a pull-it-together focus for the emerging retiree. The wholistic-based framework guides people through issues in the health, housing, financial, legal and family domains.

